# Supplementary material for: Nonenzymatic RNA copying with a potentially primordial genetic alphabet
Source: Proc Natl Acad Sci U S A. 2025 May 21;122(21):e2505720122. doi: 10.1073/pnas.2505720122 (PMC12130883; doi:10.1073/pnas.2505720122)
Supplement: Supplementary file 1 — Appendix 01 (PDF) [file pnas.2505720122.sapp.pdf]

Supplementary Information for

Nonenzymatic RNA copying with a potentially primordial genetic alphabet

Ziyuan Fang[a,1], Xiwen Jia[a,b,1], Yanfeng Xing[c] and Jack W. Szostak[a,2]

[a]Howard Hughes Medical Institute, Department of Chemistry, The University of Chicago, Chicago, Illinois 60637, United States

[b]Department of Chemistry and Chemical Biology, Harvard University, Cambridge, Massachusetts 02138, United States

[c]Department of Biochemistry and Molecular Biology, The University of Chicago, Chicago, Illinois 60637, United States

[1]Z.F. and X.J. contributed equally to this work.

[2]To whom correspondence may be addressed. **Email:** jwszostak@uchicago.edu

This PDF file includes:

Synthesis and characterization of homo-dinucleotides
Figures S1 to S4
Tables S1 to S15

1.Synthesis and characterization of homo-bridged-dinucleotides

The following 5′-5′-2-aminoimidazolium-bridged-dinucleotides (A*A, C*C, G*G, U*U, I*I, s^2C*s^2C, s^2U*s^2U) were synthesized following a previously reported procedure (1). Details of the nuclear magnetic resonance (NMR) spectra and high-resolution mass spectrometry (HRMS) are provided below.

1.1. 1,3-di-(adenosine-5'phosphoryl)-2-aminoimidazolium (A*A)

^{1}H NMR (400 MHz, D_2O) δ 8.12 (s, 2H), 8.07 (s, 2H), 6.74 – 6.71 (m, 2H), 5.87 (d, J = 4.8 Hz, 2H), 4.59 (t, J = 4.8 Hz, 2H), 4.37 (t, J = 4.9 Hz, 2H), 4.16 – 4.06 (m, 4H), 4.05 – 3.98 (m, 2H). Peaks corresponding to residual TEAB observed at 3.20 and 1.27 ppm.

^{31}P NMR (162 MHz, D_2O) δ -12.81. (The purity is 100% with no detectable activated monomer peak)

HRMS (Q-TOF) m/z: $[M - H]^-$ Calcd. for $C_{23}H_{28}N_{13}O_{12}P_2$ 740.1456; Found: 740.1575.

1.2.1 1,3-di-(cytidine-5'phosphoryl)-2-aminoimidazolium (C*C)

^{1}H NMR (400 MHz, D_2O) δ 7.64 (d, J = 7.4 Hz, 2H), 6.93 (s, 2H), 6.02 (d, J = 7.5 Hz, 2H), 5.82 (d, J = 3.2 Hz, 2H), 4.25 – 4.11 (m, 10H). Peaks corresponding to residual TEAB observed at 3.20 and 1.27 ppm.

^{31}P NMR (162 MHz, D_2O) δ -12.82. (The purity is 100% with no detectable activated monomer peak)

HRMS (Q-TOF) m/z: $[M - H]^-$ Calcd. for $C_{21}H_{28}N_9O_{14}P_2$ 692.1231; Found: 692.1348.

1.3 1,3-di-(guanosine-5'phosphoryl)-2-aminoimidazolium (G*G)

^{1}H NMR (400 MHz, D_2O) δ 7.88 (s, 2H), 6.70 – 6.68 (m, 2H), 5.75 (d, J = 5.2 Hz, 2H), 4.65 (t, J = 5.1 Hz, 2H), 4.39 (t, J = 5.0 Hz, 2H), 4.14 – 4.07 (m, 4H), 4.03 – 3.96 (m, 2H). Peaks corresponding to residual TEAB observed at 3.19 and 1.27 ppm.

^{31}P NMR (162 MHz, D_2O) δ -12.84. (The purity is 100% with no detectable activated monomer peak)

HRMS (Q-TOF) m/z: $[M - H]^-$ Calcd. for $C_{23}H_{28}N_{13}O_{14}P_2$ 772.1354; Found: 772.1491.

1.4. 1,3-di-(uridine-5'phosphoryl)-2-aminoimidazolium (U*U)

^{1}H NMR (400 MHz, D_2O) δ 7.64 (d, J = 8.4 Hz, 2H), 6.95 (t, J = 2.0 Hz, 2H), 5.88 (d, J = 7.6 Hz, 2H), 5.82 (d, J = 4.3 Hz, 2H), 4.27 (t, J = 4.9 Hz, 2H), 4.21 (t, J = 5.3 Hz, 2H), 4.19 – 4.16 (m, 4H), 4.15 – 4.11 (m, 2H). Peaks corresponding to residual TEAB observed at 3.20 and 1.27 ppm.

^{31}P NMR (162 MHz, D_2O) δ -12.87. (The purity is 100% with no detectable activated monomer peak)

HRMS (Q-TOF) m/z: $[M - H]^-$ Calcd. for $C_{21}H_{26}N_7O_{16}P_2$ 694.0932; Found: 694.1042.

1.5. 1,3-di-(inosine-5'phosphoryl)-2-aminoimidazolium (I*I)

^{1}H NMR (400 MHz, D_2O) δ 8.15 (s, 2H), 6.71 (t, *J* = 2.0 Hz, 2H), 5.90 (d, *J* = 4.9 Hz, 2H), 4.77 (s, 2H), 4.63 (t, J = 5.2 Hz, 2H), 4.40 (t, J = 4.8 Hz, 2H), 4.08-4.16 (m, 4H), 4.07-3.98 (m, 2H). Peaks corresponding to residual TEAB observed at 3.19 and 1.27 ppm.

^{31}P NMR (162 MHz, D_2O) δ -12.90. (The purity is 100% with no detectable activated monomer peak)

HRMS (Q-TOF) m/z: $[M - H]^-$ Calcd. for $C_{23}H_{26}N_{11}O_{14}P_2$ 742.1141; Found: 742.1248.

1.6. 1,3-di-(2-thiocytidine-5'phosphoryl)-2-aminoimidazolium (s^2C*s^2C)

^{1}H NMR (400 MHz, D_2O) δ 7.81 (d, J = 7.5 Hz, 2H), 7.00 (s, 2H), 6.52 (s, 2H), 6.29 (d, J = 7.6 Hz, 2H), 4.39 – 4.04 (m, 10H). Peaks corresponding to residual TEAB observed at 3.20 and 1.27 ppm.

^{31}P NMR (162 MHz, D_2O) δ -12.86. **(**The purity is 100% with no detectable activated monomer peak)

HRMS (Q-TOF) m/z: $[M - H]^-$ Calcd. for $C_{21}H_{28}N_9O_{12}S_2P_2$ 724.0780; Found: 724.0881.

1.7 1,3-di-(2-thiouridine-5'phosphoryl)-2-aminoimidazolium (s^2U*s^2U)

^{1}H NMR (400 MHz, D_2O) δ 7.75 (d, J = 8.3 Hz, 2H), 7.00 (s, 2H), 6.56 (s, 2H), 6.10 (d, J = 8.6 Hz, 2H), 4.30 – 4.18 (m, 6H), 4.18 – 4.12 (m, 4H). Peaks corresponding to residual TEAB observed at 3.19 and 1.27 ppm.

^{31}P NMR (162 MHz, D_2O) δ -12.94. (The purity is 100% with no detectable activated monomer peak)

HRMS (Q-TOF) m/z: $[M - H]^-$ Calcd. for $C_{21}H_{26}N_7O_{14}S_2P_2$ 726.0460; Found: 726.0574.

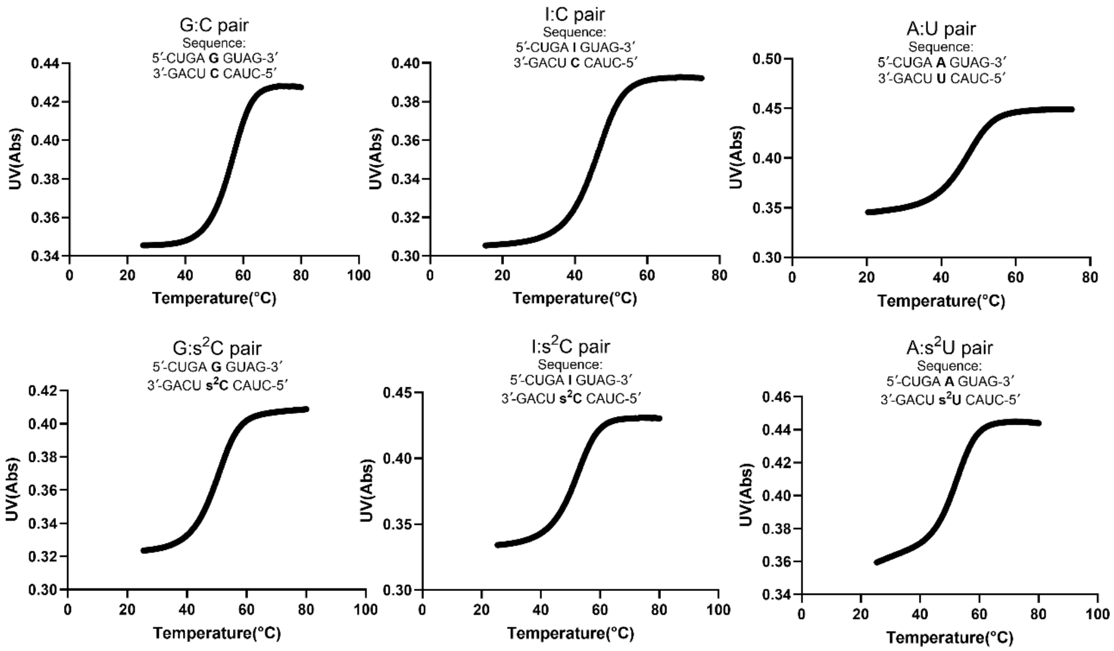

Figure S1. Representative melting curves collected during the thermal denaturation of 5 µM oligonucleotides in 10 mM Tris-HCl pH 8.0, 1 M NaCl, and 2.5 mM EDTA.

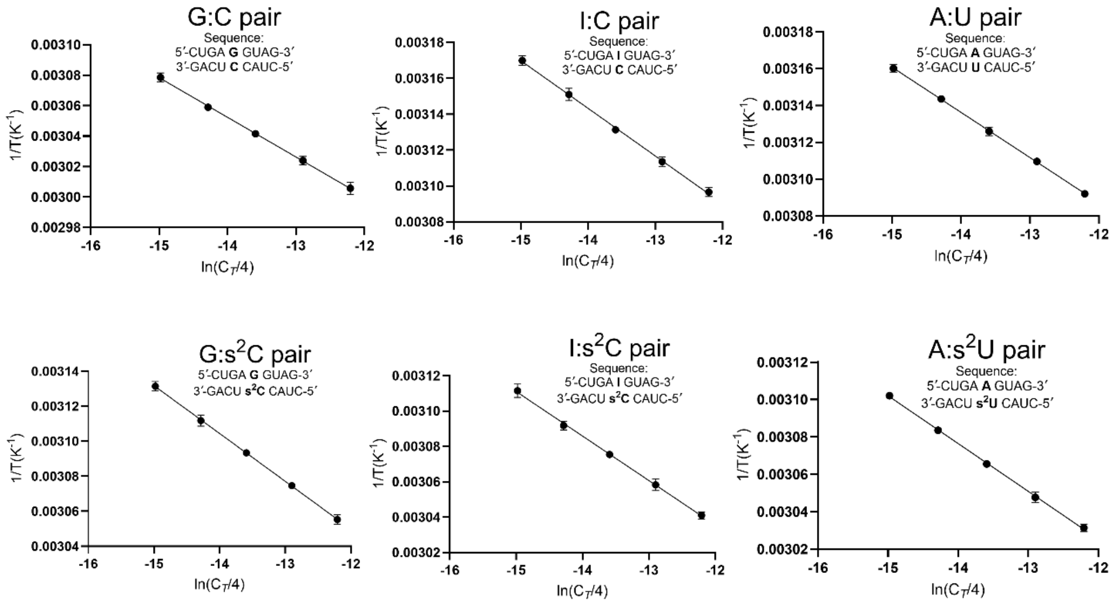

Figure S2. Linear least-squared fits of a Van’t Hoff plot of inverse melting temperature (T_m^{-1}) collected from optical melts at different oligonucleotide concentrations.

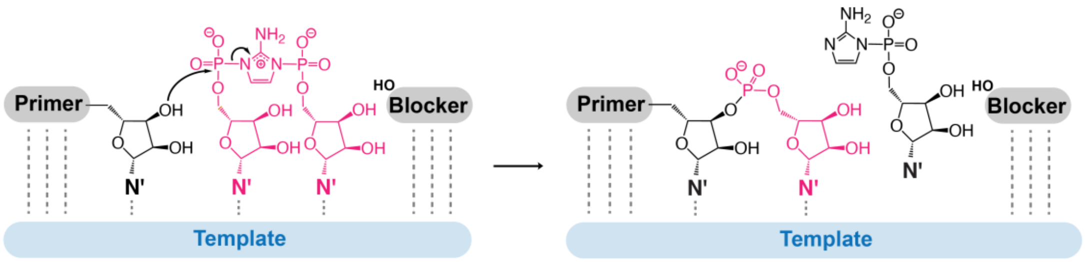

Figure S3. Mechanism of bridged dinucleotide (N*N) primer extension within the template-primer-blocker complex.

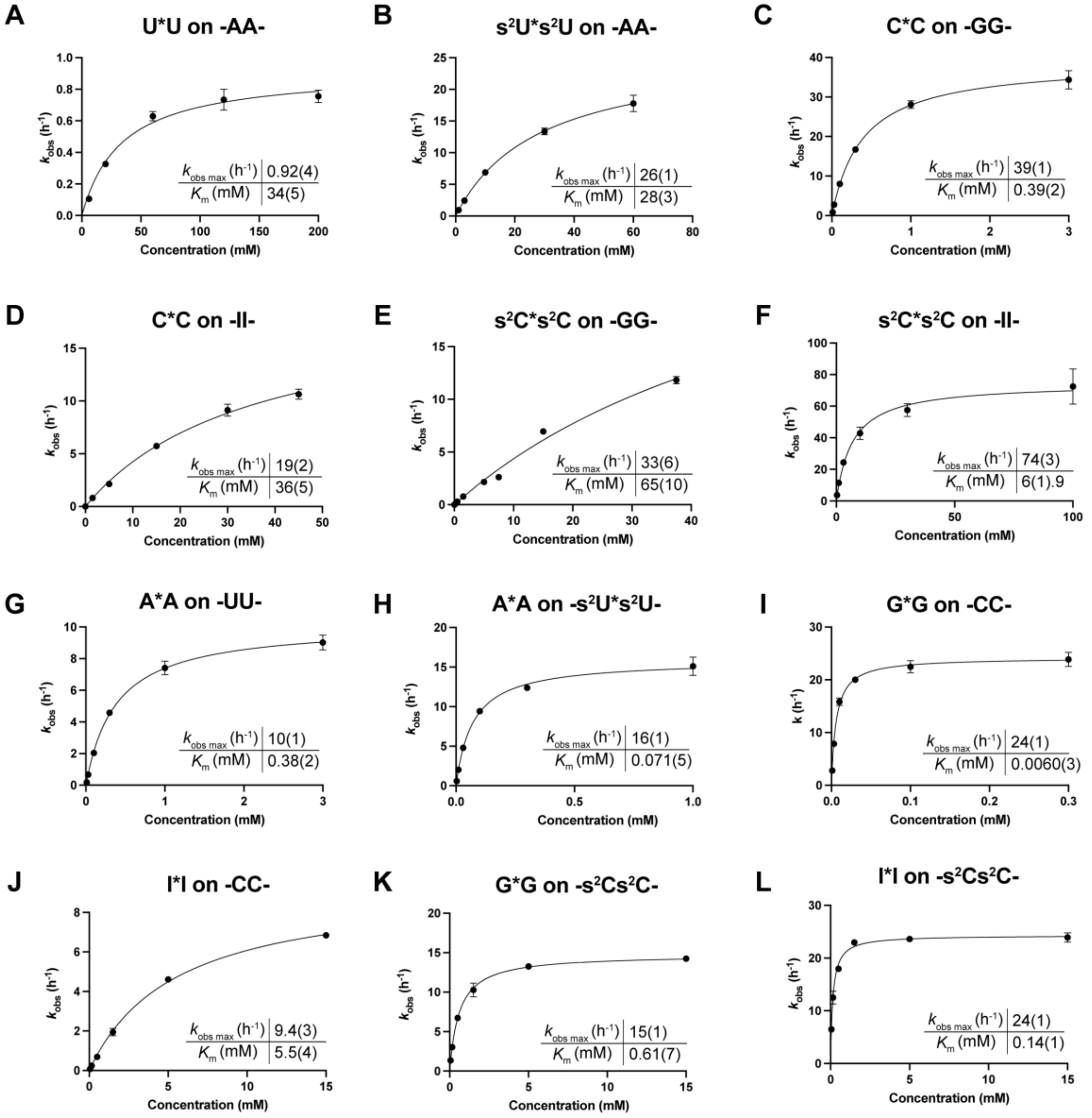

Figure S4. Michaelis Menten curves for primer extension reactions with bridged substrates on the indicated template sequences (A) U*U on -AA-, (B) s^{2}U*s^{2}U on -AA-, (C) C*C on -GG-, (D) C*C on -II- (E) s^{2}C*s^{2}C on -GG- (F) s^{2}C*s^{2}C on -II- (G) A*A on -UU- (H) A*A on -s^{2}Us^{2}U- (I) G*G on -CC- (J) I*I on -CC- (K) G*G on -s^{2}Cs^{2}C- (L) I*I on -s^{2}Cs^{2}C-

Table S1. Optimized Conditions for Crystallization

Sequence	Optimized crystallization conditions
GCS1	1.4 M Sodium citrate tribasic dihydrate, 0.1 M HEPES pH 7.5
GCS2	0.2 M Magnesium chloride hexahydrate, 0.1 M HEPES sodium pH 7.5, 30% v/v Polyethylene glycol 400
ICS1	0.2 M Calcium acetate hydrate, 0.1 M Sodium cacodylate trihydrate pH 6.5, 18% w/v Polyethylene glycol 8,000
ICS2	2.0 M Ammonium sulfate, 5% v/v 2-Propanol
Native16	20 % v/v Polyethylene glycol 200, 50 mM HEPES pH 7.5, 200 mM Potassium Chloride, 25 mM Magnesium Sulfate
AUS1	2.4 M Sodium malonate pH 7.0
AUS2	0.2 M Magnesium chloride hexahydrate, 0.1 M HEPES sodium pH 7.5, 30% v/v Polyethylene glycol 400

Table S2. Data Collection Statistics

Sequences	GCS1	GCS2	ICS1	ICS2
PDB code	9CSO	9CSP	9CSQ	9CSR
Beamline	8.2.1	8.2.1	8.2.1	8.2.1
Wavelength (Å)	1.00003	1.00003	1.00003	1.00003
Space group	*R*32	*R*32	*R*32	*R*32
Unit cell parameters (Å, °)	41.34, 41.34, 124.15, 90, 90, 120	41.22, 41.22,123.47, 90, 90, 120	43.40, 43.40, 123.14, 90, 90, 120	40.94, 40.94, 123.48, 90, 90, 120
Resolution range (Å)	50-1.40 (1.42-1.40)	50-1.60 (1.63-1.60)	41.05-1.54 (1.57-1.54)	50-1.33 (1.35-1.33)
Unique reflections	8355 (389)	5469 (228)	6923 (339)	9369(427)
Completeness (%)	99.1 (95.6)	97.5 (82.9)	99.9 (100)	98.4 (92.0)
R_{merge} (%)	4.8 (50.4)	10.6 (25.4)	5.2 (72.5)	6.8 (47.1)
<I/σ(I)>	23.4 (3.7)	14.8 (2.7)	19.0 (2.5)	21.6 (2.5)

Sequences	Native16	AUS1	AUS2
PDB code	9MDW	9MDX	9MDY
Beamline	5.0.1	5.0.1	5.0.1
Wavelength (Å)	0.97741	0.97741	0.97741
Space group	*R*32	*R*32	*R*32
Unit cell parameters (Å, °)	41.16, 41.16, 124.20, 90, 90, 120	41.38, 41.38, 124.07, 90, 90, 120	41.00, 41.00, 122.92, 90, 90, 120
Resolution range (Å)	50-1.42 (1.44-1.42)	50-1.50 (1.53-1.50)	50-1.36 (1.38-1.36)
Unique reflections	7975 (385)	6663 (323)	8888 (405)
Completeness (%)	100 (100)	96.6 (95.3)	100 (100)
R_{merge} (%)	7.1 (48.1)	5.7 (47.3)	4.4 (49.3)
<I/σ(I)>	24.3 (4.2)	34.6 (4.8)	49.4 (3.4)

Table S3. Data Refinement Statistics

Sequences	GCS1	GCS2	ICS1	ICS2
PDB code	9CSO	9CSP	9CSQ	9CSR
RNA strands per asymmetric unit	1	1	1	1
Resolution range (Å)	31.0-1.40	34.3-1.60	36.0 -1.54	30.75-1.33
Number of reflections	8258	5162	6881	9192
R_{work} (%)	18.9	17.6	22.2	18.8
R_{free} (%)	22.4	26.4	24.5	21.8
Bond length R.M.S. (Å)	2.07	2.26	2.48	2.35
Bond angle R.M.S. (°)	0.020	0.011	0.013	0.017
Average B-factors ($Å^2$)	16.3	13.5	29.1	12.9

Sequences	Native16	AUS1	AUS2
PDB code	9MDW	9MDX	9MDY
RNA strands per asymmetric unit	1	1	1
Resolution range (Å)	41.4-1.42	34.4 -1.50	41.0-1.36
Number of reflections	7944	6647	8856
R_{work} (%)	18.1	18.8	18.6
R_{free} (%)	22.0	24.1	21.8
Bond length R.M.S. (Å)	1.98	0.98	2.25
Bond angle R.M.S. (°)	0.010	0.005	0.013
Average B-factors ($Å^2$)	11.1	12.9	12.2

Table S4. Local base pair parameters for GCS1

Pair	Shear (Å)	Stretch (Å)	Stagger (°)	Buckle (°)	Propeller (°)	Opening (°)
A-U	-0.07	-0.15	0.01	-1.98	-10.58	-0.34
G-c	-0.21	-0.09	-0.34	-5.77	-17.3	-7.27
A-U	0.05	-0.13	-0.04	-2.9	-10.99	0.12
G-C	-0.37	-0.15	-0.15	-3.19	-11.15	0.21
A-U	0.05	-0.15	-0.1	-3.96	-8.29	3.18
A-U	-0.44	-0.05	-0.08	-4.9	-16.22	3.52
G-C	-0.3	-0.14	0.05	-3.46	-10.59	0.72
A-U	-0.01	-0.15	-0.12	-1.24	-14.71	0.15
U-A	0.01	-0.15	-0.12	1.24	-14.71	0.16
C-G	0.3	-0.14	0.05	3.46	-10.59	0.73
U-A	0.44	-0.05	-0.08	4.9	-16.22	3.51
U-A	-0.05	-0.15	-0.1	3.96	-8.29	3.18
C-G	0.37	-0.15	-0.15	3.19	-11.15	0.2
U-A	-0.05	-0.13	-0.04	2.9	-10.99	0.12
c-G	0.21	-0.09	-0.34	5.77	-17.3	-7.27
U-A	0.07	-0.15	0.01	1.98	-10.58	-0.34

Table S5. Local base pair step parameters for GCS1

Step	Shift (Å)	Slide (Å)	Rise (Å)	Tilt (°)	Roll (°)	Twist (°)	Overlap Area (Å^2)
AG/cU	-0.86	-1.24	3.29	-0.28	7.89	33.59	2.68
GA/Uc	0.88	-1.24	3.13	-0.92	4.44	33.33	5.51
AG/CU	-0.33	-1.42	3.24	-0.59	10.77	33.07	1.72
GA/UC	0.38	-1.53	3.27	0.14	10.48	30.41	4.78
AA/UU	0.58	-1.81	3.21	2.58	14.07	31.51	2.30
AG/CU	-0.15	-2.13	3.07	-0.86	10.5	25.21	2.30
GA/UC	-0.56	-1.53	3.17	-0.11	5.25	33.48	3.48
AU/AU	0	-1.15	3.11	0	5.56	32.22	8.33
UC/GA	0.56	-1.53	3.17	0.11	5.25	33.48	3.48
CU/AG	0.15	-2.13	3.07	0.85	10.5	25.21	2.30
UU/AA	-0.58	-1.81	3.21	-2.58	14.07	31.5	2.30
UC/GA	-0.38	-1.53	3.27	-0.14	10.48	30.41	4.78
CU/AG	0.33	-1.42	3.24	0.59	10.76	33.08	1.72
Uc/GA	-0.88	-1.24	3.13	0.92	4.45	33.32	5.51
cU/AG	0.86	-1.24	3.29	0.27	7.89	33.59	2.68

Table S6. Local base pair parameters for GCS2

Pair	Shear (Å)	Stretch (Å)	Stagger (°)	Buckle (°)	Propeller (°)	Opening (°)
A-U	0.04	-0.19	0.13	0.55	-10.19	-0.24
G-C	-0.28	-0.27	-0.07	-2.83	-14.2	-0.76
A-U	0.01	-0.21	0.08	-1.97	-11.87	1.79
G-C	-0.49	-0.23	-0.22	-2.39	-9.86	-1.56
A-U	-0.04	-0.16	-0.19	-6.94	-10.99	4.53
A-U	0.13	-0.16	-0.25	-10.74	-13.63	1.5
G-c	-0.1	-0.06	-0.24	-10.72	-12	-8.98
A-U	-0.04	-0.21	0.06	-3.1	-12.07	2.05
U-A	0.04	-0.21	0.06	3.11	-12.08	2.05
c-G	0.1	-0.06	-0.24	10.72	-12	-8.98
U-A	-0.13	-0.16	-0.24	10.73	-13.63	1.49
U-A	0.04	-0.16	-0.19	6.94	-10.99	4.53
C-G	0.49	-0.23	-0.22	2.39	-9.87	-1.57
U-A	-0.01	-0.21	0.08	1.97	-11.87	1.78
C-G	0.28	-0.27	-0.07	2.83	-14.19	-0.76
U-A	-0.04	-0.19	0.13	-0.54	-10.19	-0.24

Table S7. Local base pair step parameters for GCS2

Step	Shift (Å)	Slide (Å)	Rise (Å)	Tilt (°)	Roll (°)	Twist (°)	Overlap Area (Å^2)
AG/CU	-0.57	-1.18	3.24	-0.84	6.4	34.16	2.46
GA/UC	0.64	-1.32	3.15	-0.21	3.48	33.87	4.97
AG/CU	-0.29	-1.63	3.21	0.46	11.91	29.17	1.97
GA/UC	0.57	-1.68	3.36	-0.95	13.31	31.25	4.42
AA/UU	0.41	-1.69	3.33	2.86	12.66	32.83	2.95
AG/cU	-0.64	-1.87	3.23	1.47	8.96	27.91	1.73
GA/Uc	0.44	-1.39	3.06	-1.27	6.97	30.76	5.08
AU/AU	0	-1.08	3.08	0	9.3	31.14	8.82
Uc/GA	-0.44	-1.39	3.06	1.27	6.97	30.76	5.08
cU/AG	0.64	-1.87	3.23	-1.47	8.95	27.9	1.76
UU/AA	-0.41	-1.69	3.33	-2.86	12.66	32.84	2.95
UC/GA	-0.57	-1.68	3.36	0.95	13.31	31.24	4.42
CU/AG	0.29	-1.63	3.21	-0.46	11.91	29.17	1.97
UC/GA	-0.64	-1.32	3.15	0.21	3.48	33.87	4.97
CU/AG	0.57	-1.18	3.24	0.84	6.4	34.17	2.46

Table S8. Local base pair parameters for ICS1

Pair	Shear (Å)	Stretch (Å)	Stagger (°)	Buckle (°)	Propeller (°)	Opening (°)
A-U	0.02	0.04	-0.03	-4.75	-9.42	-2.59
I-c	-0.31	-0.23	0.04	2.83	-11.95	-3.31
A-U	0.06	-0.17	0.03	2.51	-13.32	3.06
G-C	-0.55	-0.2	-0.14	-3.68	-10.92	0.66
A-U	-0.04	-0.15	-0.09	-9.07	-5.73	1.26
A-U	0.24	-0.22	-0.1	-6.04	-9.63	-0.95
G-C	-0.12	-0.12	0.08	-6.41	-14.79	0.56
A-U	-0.08	-0.28	0.08	-0.94	-12.26	3.21
U-A	0.08	-0.28	0.08	0.94	-12.26	3.2
C-G	0.12	-0.12	0.08	6.41	-14.78	0.56
U-A	-0.24	-0.22	-0.1	6.03	-9.63	-0.95
U-A	0.04	-0.15	-0.09	9.07	-5.73	1.26
C-G	0.55	-0.2	-0.14	3.68	-10.92	0.67
U-A	-0.06	-0.17	0.03	-2.51	-13.32	3.06
c-I	0.31	-0.23	0.04	-2.83	-11.95	-3.31
U-A	-0.02	0.04	-0.03	4.75	-9.42	-2.6

Table S9. Local base pair step parameters for ICS1

Step	Shift (Å)	Slide (Å)	Rise (Å)	Tilt (°)	Roll (°)	Twist (°)	Overlap Area (Å^2)
AI/cU	-0.42	-1.16	3.01	-0.73	2	30.81	4.08
IA/Uc	0.62	-1.12	3.23	-0.68	7.79	34.78	5.12
AG/CU	-0.33	-1.69	3.25	-0.97	12.25	30.82	1.53
GA/UC	0	-1.74	3.3	-1.69	13.96	32.14	3.78
AA/UU	0.16	-1.53	3.19	2.24	8.9	32.77	3.17
AG/CU	0.47	-2.01	3.13	1.56	9.07	28.29	3.08
GA/UC	-0.28	-1.6	3.09	-0.78	5.6	30.88	3.62
AU/AU	0	-1.07	3.1	0	8.09	31.78	8.94
UC/GA	0.28	-1.6	3.09	0.78	5.6	30.88	3.62
CU/AG	-0.47	-2.01	3.13	-1.57	9.07	28.28	3.08
UU/AA	-0.16	-1.53	3.19	-2.23	8.9	32.76	3.17
UC/GA	0	-1.74	3.3	1.69	13.96	32.14	3.78
CU/AG	0.33	-1.69	3.25	0.97	12.25	30.82	1.53
Uc/IA	-0.62	-1.12	3.23	0.68	7.79	34.78	5.12
cU/AI	0.42	-1.16	3.02	0.72	1.99	30.8	4.08

Table S10. Local base pair parameters for ICS2

Pair	Shear (Å)	Stretch (Å)	Stagger (°)	Buckle (°)	Propeller (°)	Opening (°)
A-U	-0.07	-0.19	0.07	-0.78	-10.33	0.7
G-C	-0.23	-0.26	-0.05	-3.29	-13.84	-1.61
A-U	0.07	-0.16	0.02	-2.32	-13.34	2.46
G-C	-0.34	-0.22	-0.07	-1.24	-11.34	-1.55
A-U	0.05	-0.21	-0.1	-4.04	-8.17	2.39
A-U	-0.03	-0.03	-0.09	-11.06	-16.06	3.75
I-c	-0.28	-0.2	0.04	-1.61	-8.43	0.93
A-U	-0.05	-0.21	0.02	0.24	-17.28	1.38
U-A	0.05	-0.21	0.02	-0.24	-17.28	1.38
c-I	0.28	-0.2	0.04	1.61	-8.43	0.93
U-A	0.03	-0.03	-0.09	11.06	-16.06	3.75
U-A	-0.05	-0.21	-0.1	4.04	-8.17	2.39
C-G	0.34	-0.22	-0.07	1.24	-11.34	-1.55
U-A	-0.07	-0.16	0.02	2.32	-13.34	2.46
C-G	0.23	-0.26	-0.05	3.29	-13.84	-1.61
U-A	0.07	-0.19	0.07	0.78	-10.33	0.7

Table S11. Local base pair step parameters for ICS2

Step	Shift (Å)	Slide (Å)	Rise (Å)	Tilt (°)	Roll (°)	Twist (°)	Overlap Area (Å^2)
AG/CU	-0.6	-1.07	3.23	-1.08	6.88	33.94	3.13
GA/UC	0.69	-1.2	3.18	0.67	5.02	33.98	5.23
AG/CU	-0.52	-1.4	3.16	-1.82	10.96	31.88	1.90
GA/UC	0.47	-1.61	3.31	0.26	11.14	29.83	4.71
AA/UU	0.87	-1.79	3.36	3.85	15.78	31.87	3.51
AI/cU	-0.64	-1.79	2.92	-1.02	7.46	25.91	1.97
IA/Uc	-0.13	-1.33	3.2	0.03	8.03	32.79	3.32
AU/AU	0	-1.24	3.2	0	6.34	31.7	8.83
Uc/IA	0.13	-1.33	3.2	-0.03	8.03	32.79	3.32
cU/AI	0.64	-1.79	2.92	1.02	7.46	25.91	1.97
UU/AA	-0.87	-1.79	3.36	-3.85	15.78	31.87	3.51
UC/GA	-0.47	-1.61	3.31	-0.26	11.14	29.83	4.71
CU/AG	0.52	-1.4	3.16	1.82	10.96	31.88	1.90
UC/GA	-0.69	-1.2	3.18	-0.67	5.02	33.98	5.23
CU/AG	0.6	-1.07	3.23	1.08	6.88	33.94	3.13

Table S12. Local base pair parameters for native sequence Native16

Pair	Shear (Å)	Stretch (Å)	Stagger (°)	Buckle (°)	Propeller (°)	Opening (°)
A-U	-0.07	-0.12	0.05	0.18	-9.04	0.61
G-C	-0.21	-0.22	-0.13	-4.46	-14.88	-1.3
A-U	0.03	-0.09	0.03	-3.73	-12.03	0.75
G-C	-0.41	-0.18	-0.14	-2.66	-10.98	-1.84
A-U	0.07	-0.21	-0.11	-6.42	-8.83	1.77
A-U	0.12	-0.06	-0.18	-9.07	-14.32	1.15
G-C	-0.15	-0.15	0.06	-4.43	-10.57	-0.33
A-U	-0.02	-0.13	-0.02	-0.35	-12.71	0.28
U-A	0.02	-0.13	-0.02	0.35	-12.71	0.29
C-G	0.15	-0.15	0.06	4.43	-10.57	-0.33
U-A	-0.12	-0.06	-0.18	9.08	-14.31	1.15
U-A	-0.07	-0.21	-0.11	6.41	-8.83	1.77
C-G	0.41	-0.18	-0.14	2.66	-10.98	-1.85
U-A	-0.03	-0.09	0.03	3.74	-12.03	0.75
C-G	0.21	-0.22	-0.13	4.46	-14.88	-1.3
U-A	0.07	-0.12	0.05	-0.18	-9.05	0.6

Table S13. Local base pair step parameters for native sequence Native16

Step	Shift (Å)	Slide (Å)	Rise (Å)	Tilt (°)	Roll (°)	Twist (°)	Overlap Area (Å^2)
AG/CU	-0.64	-1.16	3.3	-0.6	7.58	34.43	2.72
GA/UC	0.58	-1.2	3.17	-0.09	4.29	33.88	5.22
AG/CU	-0.24	-1.5	3.2	-0.25	10.77	30.82	2.13
GA/UC	0.53	-1.65	3.38	0.07	11.31	31.19	4.57
AA/UU	0.68	-1.62	3.34	4.46	14.5	31.98	3.73
AG/CU	-0.03	-2.22	3.05	-0.39	10.57	24.81	2.38
GA/UC	-0.55	-1.4	3.15	-0.48	4.59	33.3	3.72
AU/AU	0	-1.06	3.2	0	9.96	31.64	8.54
UC/GA	0.55	-1.4	3.15	0.48	4.59	33.3	3.72
CU/AG	0.03	-2.22	3.05	0.39	10.58	24.81	2.38
UU/AA	-0.68	-1.62	3.34	-4.46	14.5	31.98	3.73
UC/GA	-0.53	-1.65	3.38	-0.07	11.31	31.19	4.57
CU/AG	0.24	-1.5	3.2	0.25	10.77	30.81	2.13
UC/GA	-0.58	-1.2	3.18	0.09	4.29	33.88	5.22
CU/AG	0.64	-1.16	3.3	0.6	7.58	34.43	2.72

Table S14. Combination of the primer, template, blocker and complementary oligonucleotides used in the Michaelis-Menten analysis of primer extension reactions.

Bridged dinucleotide	Template Sequence	Primer	Template	Blocker	Complementary DNA
U*U	-AA-	DL-30	LA-121	LA-111	dCLA-121
s^2U*s^2U	-AA-	DL-30	LA-121	LA-111	dCLA-121
C*C	-GG-	DL-30	LA-124	LA-111	dCLA-123
C*C	-II-	DL-30	IIT26	LA-111	dCLA-123
s^2C*s^2C	-GG-	DL-30	LA-124	LA-111	dCLA-124
s^2C*s^2C	-II-	DL-30	IIT26	LA-111	dCLA-123
A*A	-UU-	DL-30	LA-122	LA-111	dCLA-122
A*A	$-s^2Us^2U-$	DL-30	S2UT	LA-111	dCLA-122
G*G	-CC-	DL-30	LA-123	LA-111	dCLA-124
I*I	-CC-	DL-30	LA-123	LA-111	dCLA-123
G*G	$-s^2Cs^2C-$	DL-30	S2CT	LA-111	dCLA-123
I*I	$-s^2Cs^2C-$	DL-30	S2CT	LA-111	dCLA-123

Table S15. Sequences of oligonucleotides used in the Michaelis-Menten analysis of primer extension reactions.

Name	Role	Source	Type	Sequence (5'→ 3')
DL-30	Primer	IDT	RNA	/FAM/AGU GAG UAA CGG
LA-111	Blocker	IDT	RNA	G AUG UCA GAU AU
IIT26	Template	In-house	RNA	AU AUC UGA CAU C**II** CCG UUA CUC ACU
LA-121	Template	IDT	RNA	AU AUC UGA CAU C**AA** CCG UUA CUC ACU
dCLA-121	Complementary strand	IDT	DNA	AGT GAG TAA CGG **TT**G ATG TCA GAT AT
LA-122	Template	IDT	RNA	AU AUC UGA CAU C**UU** CCG UUA CUC ACU
dCLA-122	Complementary strand	IDT	DNA	AGT GAG TAA CGG **AA**G ATG TCA GAT AT
LA-123	Template	IDT	RNA	AU AUC UGA CAU C**CC** CCG UUA CUC ACU
dCLA-123	Complementary strand	IDT	DNA	AGT GAG TAA CGG **GG**G ATG TCA GAT AT
LA-124	Template	IDT	RNA	AU AUC UGA CAU C**GG** CCG UUA CUC ACU
dCLA-124	Complementary strand	IDT	DNA	AGT GAG TAA CGG **CC**G ATG TCA GAT AT
2SCT	Template	In-house	RNA	AU AUC UGA CAU C**s^{2}Cs^{2}C** CCG UUA CUC ACU
2SUT	Template	In-house	RNA	AU AUC UGA CAU C**s^{2}Us^{2}U** CCG UUA CUC ACU

References

1. D. Ding, L. Zhou, C. Giurgiu, J. W. Szostak, Kinetic explanations for the sequence biases observed in the nonenzymatic copying of RNA templates. *Nucleic Acids Research* **50**, 35-45 (2022).
